# Malaria

**Published:** 1908-12

**Authors:** D. H. Du Pree

**Affiliations:** Athens, Ga.


### MALARIA.*

BY D. H. DU PREE, B. S., M. D., ATHENS, GA.

I chose this subject because malaria presents some interesting problems in diagnosis, and because several of its forms have attacked me at one time and another. The differentiation between aestivo-autumal malaria and typhoid I shall attempt to emphasize, simply mentioning the pathology of the disease, and neglecting the treatment entirely. It is an old subject, but in the next few months some of us will be called on to make the diagnosis in a case of continued fever, and this is the excuse for these remarks.

We shall waive the definition of paludism and pass on to a description of the parasite, the recognition of which is the most important point in the diagnosis. As you know, in 1880, Laeran discovered this haemacytozoon, and a number of observers found it invariably associated with malaria. Later Bolgi differentiated the three varieties, and Ross, MacCallum, and Manson showed that man was infected by the bite of a mosquito, the diffinite host of the parasite.

The parasite in man attacks the red blood corpuscles, and there undergoes segmentation and sporulation, with the formation of comparatively few sexual elements.

In its early stages the parasite of tertian malaria, Plasmodium vivax (Haemamoeba vivax), is small, round and not pigmented. It is actively amoeboid and frequently assumes the shape of a signet ring. The nucleus is relatively large and clear. As it grows pigment appears in its cytoplasm in small granules. The invaded corpuscles are not changed in appearance at first, but become swollen and anaemic. As the plasmodium reaches maturity, it becomes almost as large as the red blood cell containing it; it gathers its pigment towards its center; and finally divides into fifteen or twenty segments that are identical with the small round bodies with which we began this description. The red blood cell is disintegrated, and the pigment of the parasite is scattered in the blood-stream to be ingested by phagocytes, or to lodge in the liver and spleen. The small hyaline bodies thus set free infect other corpuscles. Only a few sexual elements are formed, which are peculiar bodies and are known as microgame-

*Read before Eighth Congressional Medical Association, held at Athens, Ga., August 19, 1908.

tocytes (male) and macrogametocytes( female). This process takes about 48 hours for its completion, and the paroxysm is coincident with the segmentation and sporulation of the plasmodium. As this paroxysm occurs therefore every other day, this is known clinically as Tertain Malaria.

The parasite of quartain malaria, plasmodium malariae (haemamoeba malariae), is like P. vivax in the regularity of its cyclic development and in its general appearance. However, it is not so actively emoeboid, and its pigment granules are coarser. The infected haematocytes are shrunken and brassy in appearance. It divides into only six or eight segments, and its cycle of development lasts about 72 hours. Hence the paroxysm occurs every fourth day, and it derives the name Quartain Malaria from this fact. It is not so common as P. vivax.

A patient may receive a double infection with P. vivax and then he will develop a paroxysm with the maturation of each set—every 24 hours—Quotidian Malaria. Sometimes he may harbor two or (rarely) more sets of P. malariae. Often there is a mixed infection with vivax and malariae.

The parasite of destivo-autumnal malaria, plasmodium praecox (haematozoon falciparium), is smaller than the others, its pigment is scantier, and the corpuscle containing it is brassy in appearance and not increased in size. The later states disappear from the peripheral circulation and stay pretty closely in blood of certain internal organs, especially in the spleen and bone-marrow. After a week or ten days crescents and ovoids with pigment in their centers appear in the circulating blood, and these are characteristic of this form of malaria. They are the sexual forms of the parasite. Unlike the others it does not develop in regular cycles, and this is the cause of the irregular clinical manifestations.

This organism does not exist in nature except as a parasite in man, its intermediate host, and in the mosquito, its definite host. And the only mosquito that harbors it is the genus Anopheles. This mosquito is confined chiefly to the country, as it breeds only in puddles and sluggish streams. It flies mostly at night. It can be distinguished from culex, or the common mosquito, by the facts that its palpi are almost as long as its proboscis, so that it appears to have three proboscides; the palpi of culex are short. The wings of anopheles are mottled, of culex plain. Anopheles stands with its body at an angle to the supporting surface; culex stands with ts body parallel to the surface upon which it rests, with its hind legs drawn up to its body.

When a mosquito belonging to this genus anopheles bites a patient infected with malaria, the parasite is ingested along with the patient's blood. It invades the stomach-wall of its new host and there undergoes its sexual development. When this process is completed, the oöcyst bursts into the body-cavity, and the spores find their way to the salivary glands and ducts, and are injected into the blood of the next victim of the mosquito. So the cycle is completed, sexual in man, sexual in the mosquito.

Let us dismiss the pathology of the condition by saying that the essentials are a disintegration of the red blood corpuscles, enlargement of the spleen, toxaemia; and in severe cases, a grave anaemia, often a nephritis, accummulation of pigment in some internal organs, etc. We are more concerned here with the diagnosis.

The recognition of the regular Tertian, Quartain and Quotidian types is an easy matter, and does not often puzzle even the laity. There is the regularly recurring chill, followed by fever, which is "sweated off," after which the patient is fairly comfortable, or even apparently well if he possesses a phlegmatic temperament, until the next paroxysm.

It is the aestivo-autumnal that is so often puzzling. Its clinical manifestations may vary from a mild attack of intermittent fever to the pernicious types of chronic malaria, "black-water fever" or fulminating malaria, types that the fortunately becoming rare in this part of the world. It may simulate typhoid so closely that it is well nigh impossible to make a diagnosis without the aid of a microscope. The symptoms are irregular. There may be fever intermitting at irregular intervals. The initial chill of the paroxysm is often absent, and the fall in temperature as often by lysis as by crisis. The fever may be without intermission and may persist for two or three days or a couple of weeks, after which the patient may recover or go into a pernicious malaria. The paroxysms may anticipate. There is enlargement of the spleen, and frequently an initial bronchitis, as in typhoid. The patient *looks sick* like a typhoid. And the cases usually occur in the autumn—as the name indicates—along with typhoid. However, these cases of malaria with continued fever, usually begin with several intermittent paroxysms. Herpes labialis is more common in malaria. Intestinal symptoms are not so marked as in typhoid and the rose spots of the latter are not observed. The Widal reaction is negative in those patients who have never had typhoid, and crescents and ovoids are seen in the red blood cells. There is a secondary anaemia in both diseases, but in aestivo-autumnal malaria there is lacking the leucopenia and relative lymphocytosis characteristic of typhoid. Ehrlich's diazo reaction is positive in typhoid and negative in malaria. In cases of doubt, when the blood examination cannot be made, the therapeutic test with quinine may be resorted to. But where it can be done the examination of the blood is the surest method of differentiation. As Osler remarks, "one crescent makes a diagnosis."

Aestivo-autumnal malaria may be mistaken for general miliary tuberculosis, and vice versa, and here too, the surest method of diagnosis is the examination of the blood. The diazo reaction is positive in tuberculosis.

In the study of malaria and typhoid in the Johns Hopkins Hospital one case out of a series of 829 typhoids showed malarial parasites during the course of the disease.

The differential diagnosis between these two diseases interests me particularly because I have seen several cases of typhoid complicated with a distressing cinchonism and one case of pernicious malaria die under typhoid treatment.

308-309 Southern Mutual Building, Athens, Ga.

